# A hybrid deep learning approach for COVID-19 detection based on genomic image processing techniques

**DOI:** 10.1038/s41598-023-30941-0

**Published:** 2023-03-10

**Authors:** Muhammed S. Hammad, Vidan F. Ghoneim, Mai S. Mabrouk, Walid I. Al-atabany

**Affiliations:** 1 Biomedical Engineering Department, Helwan University, Helwan, Egypt; 2 Biomedical Engineering Department, Misr University for Science and Technology (MUST), 6th of October City, Egypt; 3 Center for Informatics Science, Nile University, Sheikh Zayed City, Egypt

**Keywords:** Biomedical engineering, Biological techniques

## Abstract

The coronavirus disease 2019 (COVID-19) pandemic has been spreading quickly, threatening the public health system. Consequently, positive COVID-19 cases must be rapidly detected and treated. Automatic detection systems are essential for controlling the COVID-19 pandemic. Molecular techniques and medical imaging scans are among the most effective approaches for detecting COVID-19. Although these approaches are crucial for controlling the COVID-19 pandemic, they have certain limitations. This study proposes an effective hybrid approach based on genomic image processing (GIP) techniques to rapidly detect COVID-19 while avoiding the limitations of traditional detection techniques, using whole and partial genome sequences of human coronavirus (HCoV) diseases. In this work, the GIP techniques convert the genome sequences of HCoVs into genomic grayscale images using a genomic image mapping technique known as the frequency chaos game representation. Then, the pre-trained convolution neural network, AlexNet, is used to extract deep features from these images using the last convolution (conv5) and second fully-connected (fc7) layers. The most significant features were obtained by removing the redundant ones using the ReliefF and least absolute shrinkage and selection operator (LASSO) algorithms. These features are then passed to two classifiers: decision trees and k-nearest neighbors (KNN). Results showed that extracting deep features from the fc7 layer, selecting the most significant features using the LASSO algorithm, and executing the classification process using the KNN classifier is the best hybrid approach. The proposed hybrid deep learning approach detected COVID-19, among other HCoV diseases, with 99.71% accuracy, 99.78% specificity, and 99.62% sensitivity.

## Introduction

Coronavirus disease 2019 (COVID-19) is a severe pandemic caused by a novel beta-coronavirus (βCoV) known as the severe acute respiratory syndrome coronavirus 2 (SARS-CoV-2) that emerged in Wuhan, China^1,2^. Globally, as of 12 December 2022, the World Health Organization reported that COVID-19 had resulted in 646.27 million confirmed cases with 6.64 million deaths^3^. Several viral epidemics have been identified during the past two decades that cause illnesses ranging from the common cold to more severe diseases. These epidemics are classified into four groups: gamma-coronavirus (γCoV), delta-coronavirus (δCoV), βCoV, and alpha-coronavirus (αCoV). The αCoV and βCoV infect mammals, whereas the δCoV and γCoV infect birds^4,5^. αCoV variants include HCoV-229E and HCoV-NL63. Meanwhile, the variants of βCoV are HCoV-OC43, HCoV-HKU1, severe acute respiratory syndrome coronavirus 1 (SARS-CoV-1), and middle east respiratory syndrome coronavirus (MERS-CoV)^6,7^.

The COVID-19 sequences share approximately 50% and 79% similarity to the MERS-CoV and SARS-CoV-1 sequences, respectively^8^. Due to their genetic similarities, distinguishing COVID-19 from other HCoV diseases is a challenging issue. Moreover, the most frequent symptoms of COVID-19 are shortness of breath, headache, myalgia, fever, and dry cough^9^, which are mostly similar to those of the common flu. As a result, it is difficult to detect COVID-19 at an early stage. Because COVID-19 spreads fast and threatens the public health system, it is critical to detect the positive cases and treat them immediately.

## Literature review

Medical imaging techniques are among the most effective methods for the automatic detection of COVID-19, as the produced computed tomography (CT) and X-ray images are processed using artificial intelligence approaches^10–18^. Feature extraction is an essential step required in the detection process of COVID-19. These features can be extracted using either manual methods or deep learning models^12–14^. Khuzani et al.^12^ used manual feature extraction methods to compute frequency and spatial features from X-ray images to construct a feature vector of 256 elements. Principal Component Analysis (PCA) was then used to select the most significant features, which were then used to train and test a multilayer perceptron (MLP) network to classify healthy, pneumonia, and COVID-19 cases. The system achieved an accuracy of 94%.

Chandra et al.^13^ presented binary and multiclass systems to classify normal, COVID-19, and pneumonia cases. The gray-level co-occurrence matrix (GLCM) and histogram of oriented gradients (HOG) methods were used to extract several features, which were then fed to the binary gray wolf optimization algorithm to select the significant ones. The systems used the majority vote of five classifiers: artificial neural network, decision trees (DT), naïve Bayes, support vector machine (SVM), and k-nearest neighbors (KNN). The binary and multiclass classification systems achieved 98.1% and 91.3% accuracy, respectively. Ozturk et al.^14^ used the gray level run length matrix, GLCM, and local binary pattern (LBP) methods to extract standard features from CT and X-ray images to build a binary system for detecting COVID-19. These features were optimized using the PCA algorithm to select the most prominent ones, which were then passed to the SVM classifier. The system resulted in an accuracy of 94.23%.

Nevertheless, the lack of manual feature extraction in deep learning and the presence of an end-to-end architecture have urged researchers to conduct additional research in this area. Recently, hybrid approaches have been used to detect COVID-19. These approaches used the pre-trained convolution neural network (CNN) models as feature extractors and the classical machine learning algorithms in the classification process^15–17^. Sethy et al.^15^ extracted deep features (DFs) from X-ray images using 13 pre-trained CNN models to detect COVID-19 among pneumonia and healthy cases. Besides the DFs, they extracted standard features using the following manual feature extraction algorithms: LBP, GLCM, and HOG. They used the SVM in the classification stage. Their results showed that the traditional classification model, which combined the LBP method and the SVM classifier, resulted in an accuracy of 93.4%. While the hybrid deep learning model, which combined the ResNet50 CNN model and the SVM classifier, achieved the best accuracy of 95.33%. Togaçar et al.^16^ presented a multiclass system to classify healthy, pneumonia, and COVID-19 cases. The MobileNetV2 and SqueezeNet CNN models were used to extract DFs from X-ray images, which were then passed to the social mimic optimization algorithm to detect the most significant features. After that, the optimized features were fed to the SVM classifier. The proposed system resulted in an accuracy of 99.27%. Turkoglu^17^ used the AlexNet model to extract DFs, which were optimized using the Relief algorithm and then fed to the SVM classifier to detect COVID-19 among healthy and pneumonia cases. The proposed model resulted in 99.18% accuracy.

The following section presents four novel approaches for detecting and controlling the spread of the COVID-19 pandemic^18–20,22^. Tai et al.^18^ presented a novel approach based on the extended reality and the internet of medical things technology for the telemedicine diagnostic of COVID-19. The approach combined deep learning algorithms, cloud computing, and virtual and augmented reality remote surgical plans to provide real-time treatment of COVID-19 patients. They collected clinical data from normal and COVID-19 patients, which were used to train a deep auxiliary classifier generative adversarial network for COVID-19 prediction. In addition, they used the copycat network to attack and steal the approach to enhance the security performance. The experimental results demonstrated that their approach outperformed the existing perception therapy techniques regarding performance and security, with an accuracy, F_1_-score, and recall of 0.92, 0.95, and 0.98, respectively.

Abdel-Basset et al.^19^ proposed a new hybrid approach based on the whale optimization algorithm and the slime mould algorithm (SMA) for extracting small regions that may contain COVID-19 using chest X-ray images. The proposed approach was tested on twelve chest images with different threshold levels from two to thirty and compared using the following algorithms: salp swarm, whale optimization, lshade, FireFly, Harris-hawks, and standard SMA. The following parameters were used to measure the effectiveness of the different algorithms: structured similarity index, signal-to-noise ratio, fitness values, and CPU time. Their findings demonstrated that the suggested hybrid approach outperformed the other algorithms regarding all evaluation parameters. Additionally, the standard SMA resulted in better performance than the other algorithms.

Gupta et al.^20^ presented a deep-learning model called COVID-WideNet based on a capsule neural network (CapNet) for detecting COVID-19 using X-ray images. CapNets are composed of a network of neurons that accepts and outputs vectors instead of the scaler values in CNN models. This characteristic enables the CapNet to learn the image features in addition to its deformation and viewing circumstances. Each capsule in CapNet comprises a group of neurons, with the output of each neuron reflecting a unique property of the same feature. The proposed approach was tested on the COVIDx dataset^21^. The efficiency of the proposed approach outperformed the state-of-the-art approaches. Besides, the approach had fewer trainable parameters, less than 20 times that of other CNN models, resulting in an efficient and fast detection of COVID-19, with an accuracy of 91%. Zafar et al.^22^ presented a system based on a neutrosophic cognitive map (NCM) to analyze the role of uncertain and indeterminate factors such as age, healthcare, and immunity in spreading the COVID-19 pandemic. The NCM is a modified version of a fuzzy cognitive map, which considers uncertain and indeterminate factors. Their system had the potential to limit the spread of the COVID-19 pandemic.

Although the previously mentioned classification systems based on medical imaging modalities are highly accurate and important in controlling the COVID-19 pandemic, they have significant drawbacks. These systems subject the patient to a high dose of radiation, which might have serious health consequences, particularly in pregnant women.

On the other hand, molecular techniques such as the reverse transcription-polymerase chain reaction (RT-PCR) tests are the gold standard methods for detecting COVID-19^23^. However, insufficient resources for conducting RT-PCR tests reduce the speed and efficiency of screening suspected cases. This is a problematic issue, especially with a large patient population. Moreover, several studies^24,25^ have demonstrated that RT-PCR tests have high false positive and false negative rates.

Besides the medical imaging modalities and molecular techniques, various studies have detected COVID-19 by extracting features from its genome sequence^26–31^. Arslan et al.^26^ presented a system to detect COVID-19, among other HCoV diseases, by extracting CpG-based features from whole genome sequences. These features were passed to the KNN classifier, which was implemented using several distance matrices. The KNN classifier with the L1 distance metric achieved the best accuracy of 98.8%. Furthermore, Arslan^27^ improved the previous system by combining similarities features with CpG-based features, which were used to train and test six classifiers: SVM, AdaBoost, MLP, KNN, DT, and random forest. Their experimental results showed that the system accuracy increased to 99.8%. Lopez-Rincon et al.^28^ proposed a CNN model to detect COVID-19 using whole genome sequences. The CNN model detected the subsequences with a length of 21 base pairs. They obtained 3827 features using 553 HCoV sequences and their system achieved an accuracy of 98.73%.

Saha et al.^29^ proposed a recurrent neural network, COVID-DeepPredictor, based on long-short term memory to classify COVID-19, MERS-CoV, SARS-CoV-1, and Influenza cases. They used the *k*-mer technique to divide the whole genome sequences into descriptors of sequences of length k, which were then used to train and test the COVID-DeepPredictor model. Their results showed that the COVID-DeepPredictor outperformed the other state-of-the-art prediction techniques with an accuracy above 99.51%. Harikrishnan et al.^30^ presented a neurochaos learning (NL) architecture, ChaosFEX + SVM, to classify COVID-19 and SARS-COV-1 cases. The NL uses chaotic neurons instead of dumb neurons used in traditional networks. They used the NL model to extract chaos features, which were then used to train and test the SVM classifier. The proposed approach resulted in an average accuracy of 0.998. Gomes et al.^31^ presented a new approach for enhancing the molecular diagnosis of COVID-19 by combining the results of RT-PCR tests with pseudo-convolutional machines, which were used to extract DFs from whole genome sequences. The most significant features were selected using several optimization techniques, which were then passed to four classifiers: RF, SVM, NB, and SVM. The MLP classifier outperformed the other classifiers with a sensitivity and specificity of 97 and 99%, respectively, when comparing COVID-19 with virus families with similar symptoms.

Recently, genomic signal processing (GSP) techniques have been used to detect COVID-19^32–35^. These techniques transform the genome sequences into genomic signals using various genomic signal mapping approaches. Then, these signals are processed using digital signal processing tools to build valuable systems that can detect COVID-19. Naeem et al.^32^ proposed a GSP system to classify COVID-19, SARS, and MERS diseases. They used different manual feature extraction methods to extract several features from the genomic signals, which were passed to KNN and trainable cascade-forward backpropagation network models. Their results showed that the KNN classifier achieved the best accuracy of 100%. Randhawa et al.^33^ presented a GSP system to detect COVID-19 under three genera: αCoV, βCoV, and δCoV, using six supervised machine learning algorithms. The linear discriminant analysis algorithm resulted in 100% accuracy among the different classification algorithms.

Khodaei et al.^34^ used the Z-curve technique to transform the whole genome sequences into genomic signals to classify COVID-19 and influenza cases. The Z-curve transforms the genome sequence from the nucleotides into three signal vectors (X, Y, and Z) based on the vector of each nucleotide in the sequence (A, T, C, and G). The linear predictive coding model was used to extract significant features from each signal vector, which were then fed to four classifiers: SVM with several kernels, KNN, MLP, and DT. The SVM with a sigmoid kernel achieved higher performance than the other classifiers, with an accuracy of 99.4%. Singh et al.^35^ built a GSP system based on the electron–ion interaction pseudopotential mapping technique (EIIP) to detect COVID-19, among other HCoV diseases using partial and whole genome sequences. They extracted several features from the genome signals: singular value decomposition, average and peak-to-signal noise ratio of the magnitude spectrum, average magnitude difference function, and time-domain periodogram. The most significant features were selected using the correlation-based feature selection and Pearson correlation coefficient. These features were passed to four classifiers: KNN, SVM, DT, and RF. The RF classifier outperformed the other classifiers, with an accuracy of 97.4%.

In this study, genomic image processing (GIP) techniques are rather used to detect COVID-19. GIP is a bioinformatics branch that links bioinformatics and image-processing approaches. It uses various genomic image mapping techniques^36,37^ to transform the genome sequences of HCoV diseases into genomic images. Then, it processes these images using digital image processing tools to build systems that can detect COVID-19. Hammad et al.^38^ presented a multiclass classification system to classify COVID-19, SARS-CoV-1, and MERS-CoV. The single gray-level representation (SGLR) technique was used to convert the whole genome sequences into genomic images. First-order features were extracted from the genomic images, which were then used to train and test KNN and SVM classifiers. The results demonstrated that both classifiers achieved an accuracy of 100%. However, the KNN classifier is preferred because it outperformed the SVM regarding execution time. Hammad et al.^39^ extended the previous research by transforming the whole and partial genome sequences of the seven variants of HCoV diseases into genomic grayscale images. Several standard features were extracted from these images and fed to four classifiers: NB, KNN, linear SVM, and gaussian SVM. The KNN classifier achieved an accuracy of 99.39% for detecting COVID-19, among other HCoV diseases. Table 1 presents the literature review results.

**Table 1:** Results of the literature review.

Study	Best technique	Dataset	Maximum accuracy (%)
^12^	Spatial and frequency domain features PCA feature selection algorithm MLP classifier	COVID-19: 140 Healthy: 140 Pneumonia: 140	94
^13^	Histogram of oriented gradients and GLCM features Binary gray wolf optimization SVM, naïve Bayes, KNN, decision trees, and artificial neural network classifiers	COVID-19: 434 Healthy: 19 Pneumonia: 89	Binary: 98.1 Multi: 91.3
^14^	Local binary pattern, GLCM, and gray-level run-length matrix features PCA feature selection algorithm SVM classifier	COVID-19: 101 Others: 25	94.23
^15^	ResNet50 model SVM classifier	COVID-19: 127 Healthy: 127 Pneumonia: 127	95.33
^16^	MobileNetV2 and SqueezeNet CNN models Social Mimic optimization algorithm SVM classifier	COVID-19: 295 Healthy: 65 Pneumonia: 98	99.27
^17^	AlexNet model Relief algorithm SVM classifier	COVID-19: 219 Healthy: 1583 Pneumonia: 4290	99.18
^18^	Whale optimization algorithm Slime mould algorithm	12 chest images	–
^19^	Extended reality and internet of medical things technology	COVID-19: 347 Other: 2270	92
^20^	Capsule network	COVIDx	91
^26^	CpG features KNN classifier	COVID-19: 1000 HCoV-HKU1: 18 HCoV-NL63: 61 MERS-CoV: 258 βCoV: 140 HCoV-229E: 27	98.4
^27^	CpG and similarity features KNN classifier	COVID-19: 1000 HCoV-HKU1: 27 HCoV-NL63: 64 MERS-CoV: 339 HCoV-OC43: 145 SARS-CoV-1: 12 HCoV-229E: 28	99.8
^28^	Convolution neural network model	COVID-19: 66 Others: 487	98.73
^29^	Recurrent neural network	COVID-19: 680 Influenza: 8576	99.51
^30^	Chaos features SVM classifier	COVID-19: 4498 SARS-COV-1:101	99
^31^	Deep features MLP classifier	COVID-19:171 Influenza: 347,162	98
^32^	Standard features extracted from genomic signals KNN classifier	SARS-CoV-1: 76 MERS-CoV: 76 COVID-19: 76	100
^33^	DFT features extracted from genomic signals Linear discriminant analysis classifier	COVID-19: 29 αCoV: 20 βCoV: 20 δCoV: 20	100
^34^	Linear predictive coding model SVM classifier	COVID-19:47,200 Influenza: 59,800	99.4
^35^	Standard features extracted from genomic signals Random forest classifier	COVID-19: 615 Coronavirus: 967	97.4
^38^	Standard features extracted from single nucleotide gray-level representation images KNN classifier	SARS-CoV-1: 57 MERS-CoV: 258 COVID-19: 300	100
^39^	Standard features extracted from frequency chaos game representation images KNN classifier	COVID-19: 3700 Coronavirus: 3663	99.39

*PCA* principal component analysis, *MLP* multilayer perceptron classifier, *GLCM* gray level co-occurrence matrix, *SVM* support vector machine, *KNN* K-nearest neighbors, *DFT* discrete Fourier transform.

## Main contribution

The previous COVID-19 studies^26–34,38^ had limitations related to the analysis of partial genome sequences of HCoV diseases. For instance, if a partial COVID-19 sequence is fed to the system, the system cannot predict whether the patient has COVID-19 or not. However, such a system works well with only whole COVID-19 sequences. Moreover, their datasets excluded some variants of HCoV sequences that are similar to COVID-19. To avoid these limitations in this study, an effective hybrid deep learning approach is presented. The proposed approach is based on GIP techniques to detect COVID-19, among other HCoV diseases using both whole and partial genome sequences of the seven HCoV variants. The hybrid approach employs the AlexNet model as a feature extractor along with KNN and DT classifiers in the classification process.

The main contributions adopted in this research are summarized as follows: First, transforming the whole and partial genome sequences of HCoV diseases into uniform-size grayscale images. This step is implemented using the frequency chaos game representation (FCGR) technique. Second, extracting DFs from these genome images using AlexNet CNN model instead of using manual feature extraction methods. To our knowledge, none of the previous COVID-19 studies have extracted DFs from the FCGR images in the detection of COVID-19. Third, minimization of the DFs utilizing ReliefF and least absolute shrinkage and selection operator (LASSO) algorithms. Fourth, constructing a large dataset comprising almost all variants of HCoV sequences that are similar to COVID-19 with both whole and partial genome sequences. Thus, demonstrating the effectiveness and strength of the suggested approach. This would make the proposed hybrid deep learning approach capable of distinguishing COVID-19 from other HCoV diseases. Not only achieving high accuracy but also avoiding the limitations and drawbacks of traditional and previously studied detection techniques.

## Materials and methods

**Figure 1:**
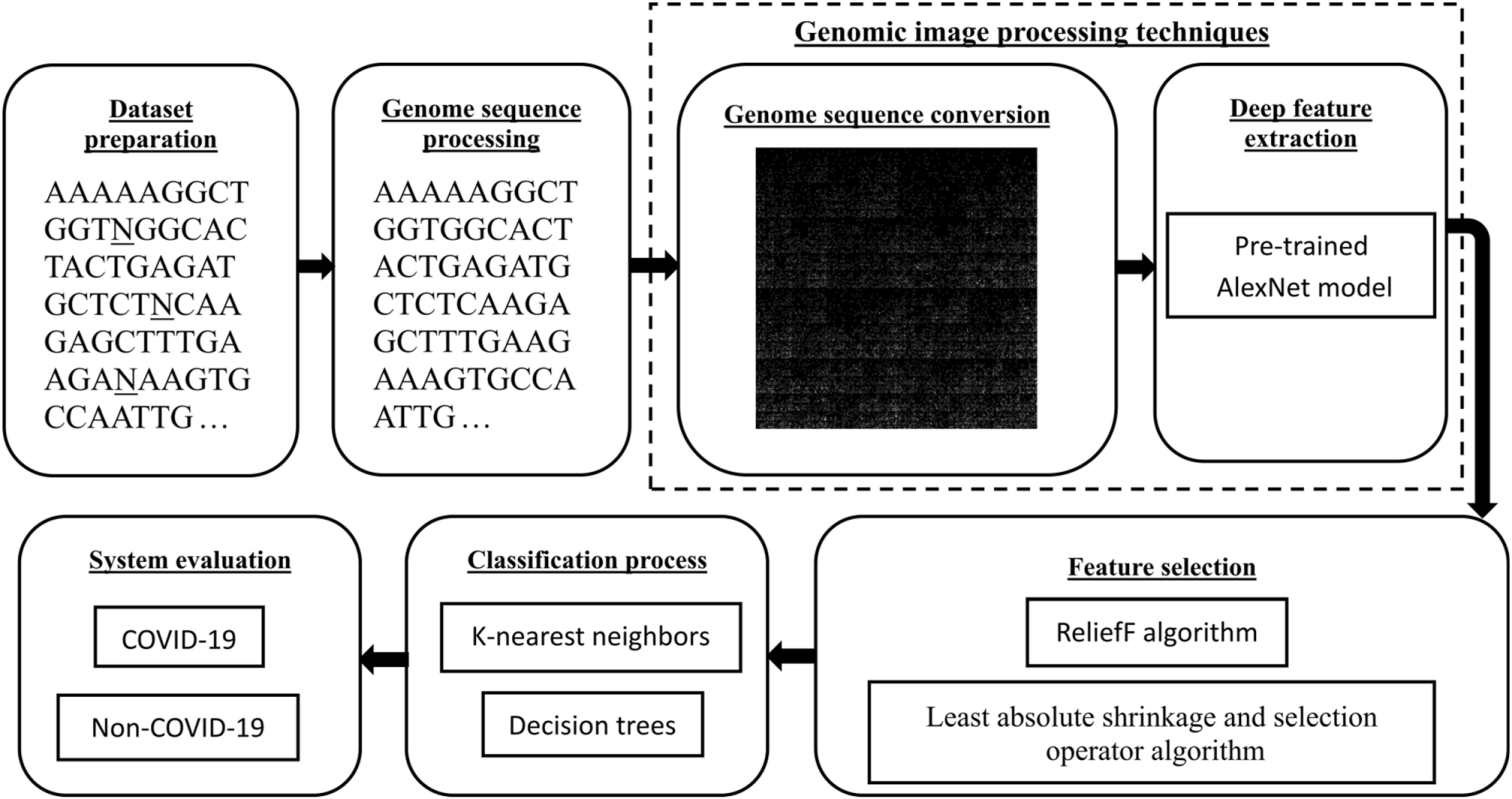
The proposed system block diagram.

The proposed hybrid deep learning system consists of seven main phases: dataset preparation, genome sequence processing and conversion, DF extraction and selection, classification, and evaluation of the proposed system. In the first phase, the HCoV sequences were downloaded. In the second phase, the downloaded sequences were analyzed to eliminate the ambiguous nucleotides (e.g., N bases), leaving only the basic nucleotides (A, T, C, and G bases). The third phase transforms the analyzed sequences into grayscale images using a genomic image mapping approach called FCGR. In the fourth phase, concerning the extraction of DFs from the FCGR images, AlexNet CNN model was employed in this investigation. ReleifF and LASSO algorithms were used to select the most significant features, which were then used to train and test KNN and DT classifiers in phases five and six, respectively. Finally, several effective parameters were used to evaluate the performance of the proposed system in the seventh phase. Figure 1 presents the block diagram of the proposed hybrid deep learning system.

### Dataset preparation

This study used whole and partial genome sequences of the seven HCoV diseases presented in Table 2. The HCoV sequences were obtained from the National Center for Biotechnology Information website^40^. All available whole and partial HCoV sequences other than COVID-19 were downloaded and used in the current study.

**Table 2:** Properties of human coronavirus sequences.

Coronavirus sequences	Class	Number of genome sequences
COVID-19 (SARS-CoV-2)	COVID-19	3700
HCoV-HKU1	Non-COVID-19	412
HCoV-NL63		637
MERS-CoV		734
HCoV-OC43		1351
SARS-CoV-1		64
HCoV-229E		465

### Genome sequence processing

In this phase, the downloaded genome sequences were analyzed to eliminate the ambiguous nucleotides (e.g., N bases), leaving only the fundamental nucleotides (A, T, C, and G bases).

### Genome sequence conversion

#### Frequency chaos game representation matrix

The FCGR technique generates a two-dimensional matrix (FCGR matrix) comprising the frequency of *k*-mers retrieved from genome sequences^41,42^. The term *k*-mer refers to the subsequences of length *k* in a given sequence. For instance, the sequence GTACAT had one 6-mer (GTACAT), two 5-mers (GTACA and TACAT), three 4-mers (GTAC, TACA, and ACAT), four 3-mers (GTA, TAC, ACA, and CAT), five 2-mers (GT, TA, AC, CA, and AT), and six 1-mers (G, T, A, C, A, T). For example, in the given sequence GTACAT, the frequency of 1-mers is A = 2, T = 2, C = 1, and G = 1.

Wang et al.^42^ showed that the FCGR matrix could be derived from the genome sequence by estimating the frequency of each *k*-mer in the genome sequence and then placing it into an appropriate location in the FCGR matrix using the following algorithm: First, the FCGR matrix is divided into four quadrants (pixels), with C, G, A, and T bases in the top left, top right, bottom left, and bottom right, respectively, to find the first-order FCGR matrix. Then, each pixel is recursively subdivided using the same approach for the kth-order FCGR matrix. Therefore, the first-order FCGR matrix (*k* = 1) is given by the following equation^42^:

1 $${\varvec{FCGR}}_{{\varvec{1}}} ({\varvec{S}}) = \left( {\begin{array}{*{20}c} {f_{C} } & {f_{G} } \\ {f_{A} } & {f_{T} } \\ \end{array} } \right),$$

where ${\varvec{F}}{\varvec{C}}{\varvec{G}}{{\varvec{R}}}_{1}\left({\varvec{S}}\right)$ represents the first-order FCGR matrix and *f *is the frequency of each 1-mer (A, T, C, and G) in the genome sequence (S).

Moreover, each pixel of the first-order FCGR matrix (A, T, C, and G of Eq. (1)) is subdivided using the previous algorithm to find the second-order FCGR matrix (*k* = 2). For instance, the pixel of A base of Eq. (1) is subdivided into four pixels with C, G, A, and T bases in the top left, top right, bottom left, and bottom right, respectively, as shown in Eq. (2). The same procedure is applied to the other pixels (T, C, and G). Therefore, the second-order FCGR matrix (*k *= 2) is given by the following equation^42^:

2 $${\varvec{FCGR}}_{2}\left({\varvec{S}}\right)= \left(\begin{array}{c}\begin{array}{cc}{f}_{CC}& {f}_{GC}\end{array} \begin{array}{cc}{f}_{CG}& {f}_{GG}\end{array}\\ \begin{array}{cc}{f}_{AC}& {f}_{TC}\end{array} \begin{array}{cc}{f}_{AG}& {f}_{TG}\end{array}\\ \begin{array}{c}\begin{array}{cc}{f}_{CA}& {f}_{GA}\end{array} \begin{array}{cc}{f}_{CT}& {f}_{GT}\end{array}\\ \begin{array}{cc}{f}_{AA}& {f}_{TA}\end{array} \begin{array}{cc}{f}_{AT}& {f}_{TT}\end{array}\end{array}\end{array}\right),$$

where ${\varvec{F}}{\varvec{C}}{\varvec{G}}{{\varvec{R}}}_{2}\left({\varvec{S}}\right)$ represents the second-order FCGR matrix and *f* is the frequency of each 2-mer (AA, TA, CA, etc.) in the genome sequence (S).

From Eqs. (1) and (2), we can see that the $${\varvec{F}}{\varvec{C}}{\varvec{G}}{{\varvec{R}}}_{{\varvec{k}}}\left({\varvec{S}}\right)$$ can be obtained from the $${\varvec{F}}{\varvec{C}}{\varvec{G}}{{\varvec{R}}}_{{\varvec{k}}-1}\left({\varvec{S}}\right)$$ by simply replacing each element ($${x})$$ in the $${\varvec{F}}{\varvec{C}}{\varvec{G}}{{\varvec{R}}}_{{\varvec{k}}-1}\left({\varvec{S}}\right)$$ by the four bases, where A, T, C, and G are in the same locations as follows: $$\left(\begin{array}{cc}{f}_{Cx}& {f}_{Gx}\\ {f}_{Ax}& {f}_{Tx}\end{array}\right).$$ Therefore the $${\varvec{F}}{\varvec{C}}{\varvec{G}}{{\varvec{R}}}_{2}\left({\varvec{S}}\right)$$ can be obtained from the $${\varvec{F}}{\varvec{C}}{\varvec{G}}{{\varvec{R}}}_{1}\left({\varvec{S}}\right)$$ using the following equation:

3 $${\varvec{FCGR}}_{2}\left({\varvec{S}}\right)= \left(\begin{array}{cc}{f}_{Cx}& {f}_{Gx}\\ {f}_{Ax}& {f}_{Tx}\end{array}\right)=\left(\begin{array}{c}\begin{array}{cc}{f}_{CC}& {f}_{GC}\end{array} \begin{array}{cc}{f}_{CG}& {f}_{GG}\end{array}\\ \begin{array}{cc}{f}_{AC}& {f}_{TC}\end{array} \begin{array}{cc}{f}_{AG}& {f}_{TG}\end{array}\\ \begin{array}{c}\begin{array}{cc}{f}_{CA}& {f}_{GA}\end{array} \begin{array}{cc}{f}_{CT}& {f}_{GT}\end{array}\\ \begin{array}{cc}{f}_{AA}& {f}_{TA}\end{array} \begin{array}{cc}{f}_{AT}& {f}_{TT}\end{array}\end{array}\end{array}\right),$$

where ${\varvec{F}}{\varvec{C}}{\varvec{G}}{{\varvec{R}}}_{2}\left({\varvec{S}}\right)$ represents the second-order FCGR matrix, and *x* represents the elements of the ${\varvec{F}}{\varvec{C}}{\varvec{G}}{{\varvec{R}}}_{1}\left({\varvec{S}}\right).$

Therefore, the third-order FCGR matrix can be estimated by replacing each element in the second-order FCGR matrix with the four bases, as shown in Eq. (4). The higher-order FCGR matrices can be computed using the same concept.

4 $${\varvec{FCGR}}_{3}\left({\varvec{S}}\right)= \left(\begin{array}{cc}{f}_{Cx}& {f}_{Gx}\\ {f}_{Ax}& {f}_{Tx}\end{array}\right)=\left(\begin{array}{cc}\begin{array}{c}\begin{array}{cc}{f}_{CCC}& {f}_{GCC}\end{array} \begin{array}{cc}{f}_{CGC}& {f}_{GGC}\end{array}\\ \begin{array}{cc}{f}_{ACC}& {f}_{TCC}\end{array} \begin{array}{cc}{f}_{AGC}& {f}_{TGC}\end{array}\\ \begin{array}{c}\begin{array}{cc}{f}_{CAC}& {f}_{GAC}\end{array} \begin{array}{cc}{f}_{CTC}& {f}_{GTC}\end{array}\\ \begin{array}{cc}{f}_{AAC}& {f}_{TAC}\end{array} \begin{array}{cc}{f}_{ATC}& {f}_{TTC}\end{array}\end{array}\end{array}& \begin{array}{c}\begin{array}{cc}{f}_{CCG}& {f}_{GCG}\end{array} \begin{array}{cc}{f}_{CGG}& {f}_{GGG}\end{array}\\ \begin{array}{cc}{f}_{ACG}& {f}_{TCG}\end{array} \begin{array}{cc}{f}_{AGG}& {f}_{TGG}\end{array}\\ \begin{array}{c}\begin{array}{cc}{f}_{CAG}& {f}_{GAG}\end{array} \begin{array}{cc}{f}_{CTG}& {f}_{GTG}\end{array}\\ \begin{array}{cc}{f}_{AAG}& {f}_{TAG}\end{array} \begin{array}{cc}{f}_{ATG}& {f}_{TTG}\end{array}\end{array}\end{array}\\ \begin{array}{c}\begin{array}{cc}{f}_{CCA}& {f}_{GCA}\end{array} \begin{array}{cc}{f}_{CGA}& {f}_{GGA}\end{array}\\ \begin{array}{cc}{f}_{ACA}& {f}_{TCA}\end{array} \begin{array}{cc}{f}_{AGA}& {f}_{TGA}\end{array}\\ \begin{array}{c}\begin{array}{cc}{f}_{CAA}& {f}_{GAA}\end{array} \begin{array}{cc}{f}_{CTA}& {f}_{GTA}\end{array}\\ \begin{array}{cc}{f}_{AAA}& {f}_{TAA}\end{array} \begin{array}{cc}{f}_{ATA}& {f}_{TTA}\end{array}\end{array}\end{array}& \begin{array}{c}\begin{array}{cc}{f}_{CCT}& {f}_{GCT}\end{array} \begin{array}{cc}{f}_{CGT}& {f}_{GGT}\end{array}\\ \begin{array}{cc}{f}_{ACT}& {f}_{TCT}\end{array} \begin{array}{cc}{f}_{AGT}& {f}_{TGT}\end{array}\\ \begin{array}{c}\begin{array}{cc}{f}_{CAT}& {f}_{GAT}\end{array} \begin{array}{cc}{f}_{CTT}& {f}_{GTT}\end{array}\\ \begin{array}{cc}{f}_{AAT}& {f}_{TAT}\end{array} \begin{array}{cc}{f}_{ATT}& {f}_{TTT}\end{array}\end{array}\end{array}\end{array}\right),$$

where ${\varvec{FCGR}}_{3}\left({\varvec{S}}\right)$ represents the third-order FCGR matrix, and *x* represents the elements of the ${\varvec{FCGR}}_{2}\left({\varvec{S}}\right)$***.***

#### Frequency chaos game representation image

We used the frequencies of the FCGR matrix to create a grayscale image with various gray levels ranging from white (most frequent) to black (least frequent). The dimension of the FCGR image is 2^*k*^ × 2^*k*^. For instance, if *k* = 8, the image dimension will be 2^8^ × 2^8^ = 256 × 256 pixels. Thus, to transform a COVID-19 sequence into a second-order FCGR image, the steps of the transformation process are as follows: First, the frequency of each 2-mer is estimated by counting the number of occurrences of each 2-mer in the genome sequence. Then, these frequencies are normalized between 0 and 255 to create a grayscale image with various gray levels ranging from white (most frequent) to black (least frequent) using Eq. (5)^43^.

5 $${{\varvec{n}}}_{{\varvec{k}}}=\frac{{{\varvec{f}}}_{{\varvec{k}}}-min}{max-min}\times 255,$$

where ${{\varvec{n}}}_{{\varvec{k}}}$ and ${{\varvec{f}}}_{{\varvec{k}}}$ represent the normalized and original frequencies of each *k*-mer, respectively, and *max* and *min* are the maximum and minimum frequencies in the genome sequence, respectively.

**Figure 2:**
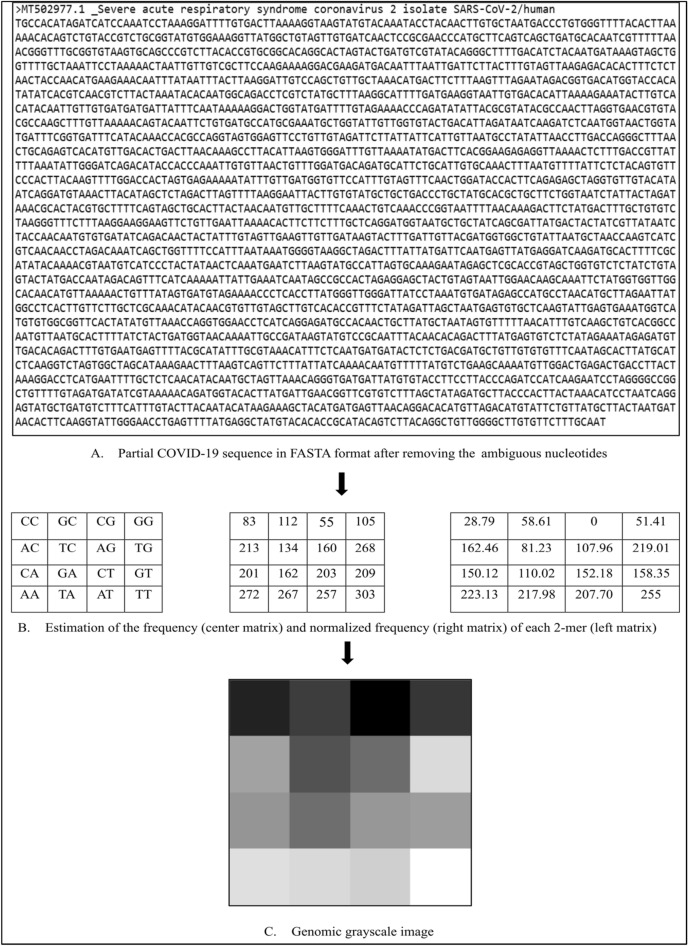
Second-order FCGR image for a COVID-19 sequence.

The set of the 2-mer frequencies of the genome sequence shown in Fig. 2A is {‘AA’: 272, ‘AC’: 213, ‘AG’: 160, ‘AT’: 257, ‘TA’: 267, ‘TC’: 134, ‘TG’: 268, ‘TT’: 303, ‘CA’: 201, ‘CC’: 83, ‘CG’: 55, ‘CT’: 203, ‘GA’: 162, ‘GC’: 112, ‘GG’: 105, ‘GT’: 209}, as shown in Fig. 2B (center matrix). The frequency range of these frequencies is 55–303, although the required range is 0–255 (minimum and maximum gray levels). Thus, the process entails subtracting 55 from each frequency, resulting in a range of 0–248. Next, each frequency is multiplied by 255/248, resulting in a range of 0–255. Therefore, the set of the normalized 2-mer frequencies of the genome sequence will be {‘AA’: 223.13, ‘AC’: 162.46, ‘AG’: 107.97, ‘AT’: 207.70, ‘TA’: 217.98, ‘TC’: 81.23, ‘TG’: 219.01, ‘TT’: 255, ‘CA’: 150.12, ‘CC’: 28.79, ‘CG’: 0, ‘CT’: 152.18, ‘GA’: 110.02, ‘GC’: 58.61, ‘GG’: 51.41, ‘GT’: 158.35}, as shown in Fig. 2B (right matrix). These normalized frequencies represent the gray levels of the genomic grayscale image, as shown in Fig. 2C.

**Figure 3:**
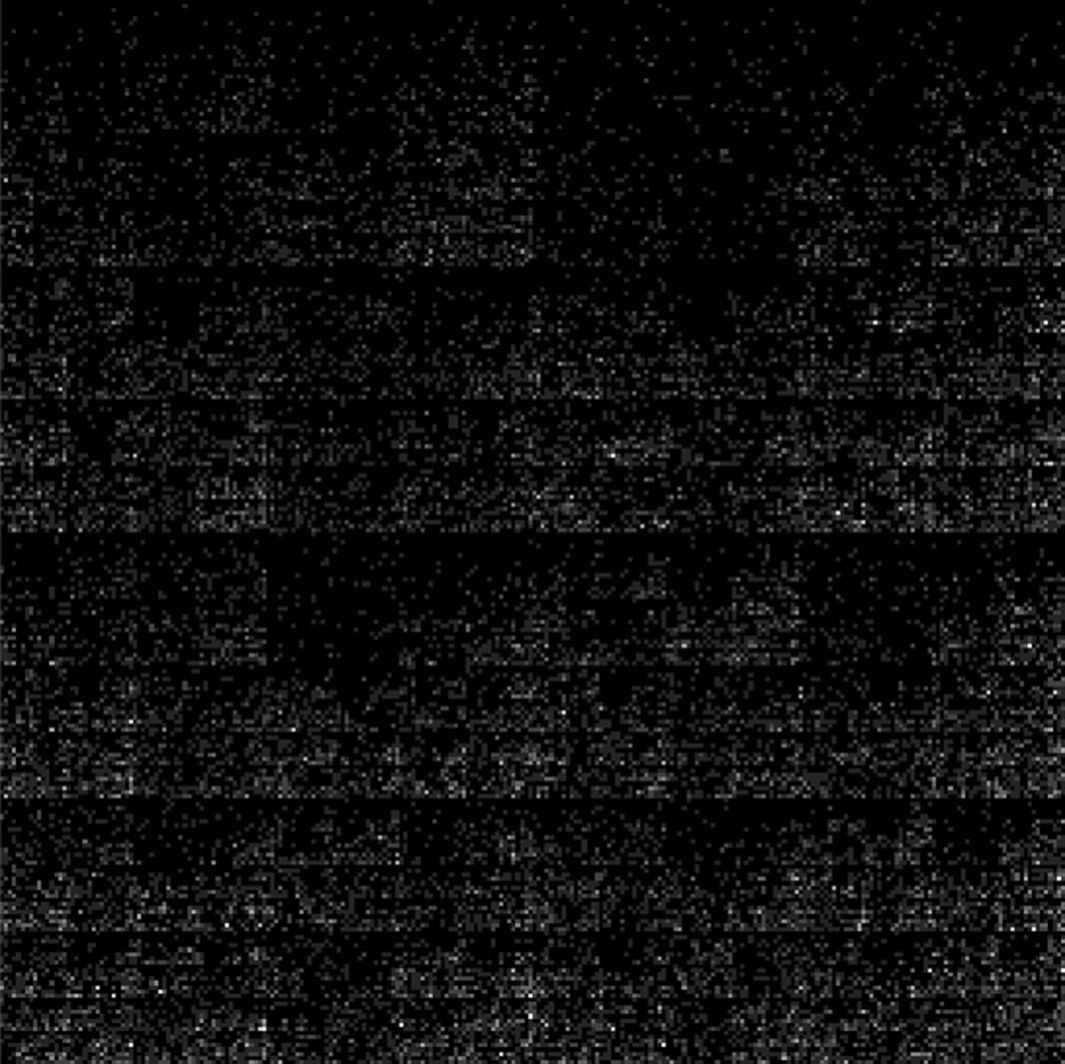
Eighth-order FCGR image for a COVID-19 sequence.

Many experiments were conducted throughout this research to evaluate the efficiency of the AlexNet model in extracting DFs from the eighth-order FCGR images to identify COVID-19. The eighth order was selected because it provides high-resolution images (256 × 256 pixels) compatible with the pre-trained CNN models, which require the image size to be at least 224 × 224 pixels. Figure 3 shows the eighth-order genomic grayscale image created using the FCGR technique for a COVID-19 sequence.

### Deep feature extraction

#### AlexNet model

**Figure 4:**
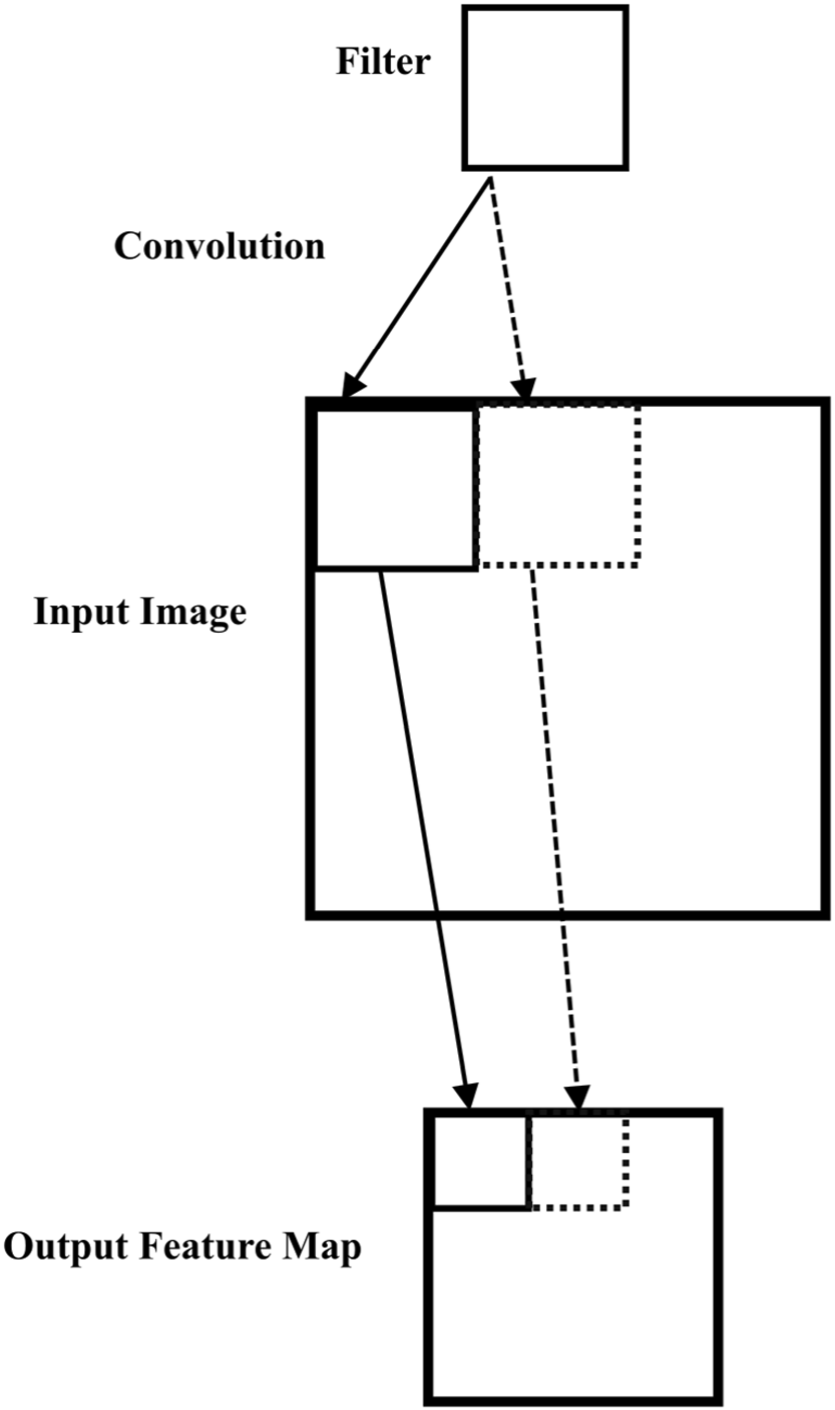
Output of convolution between an input image and a filter.

**Figure 5:**
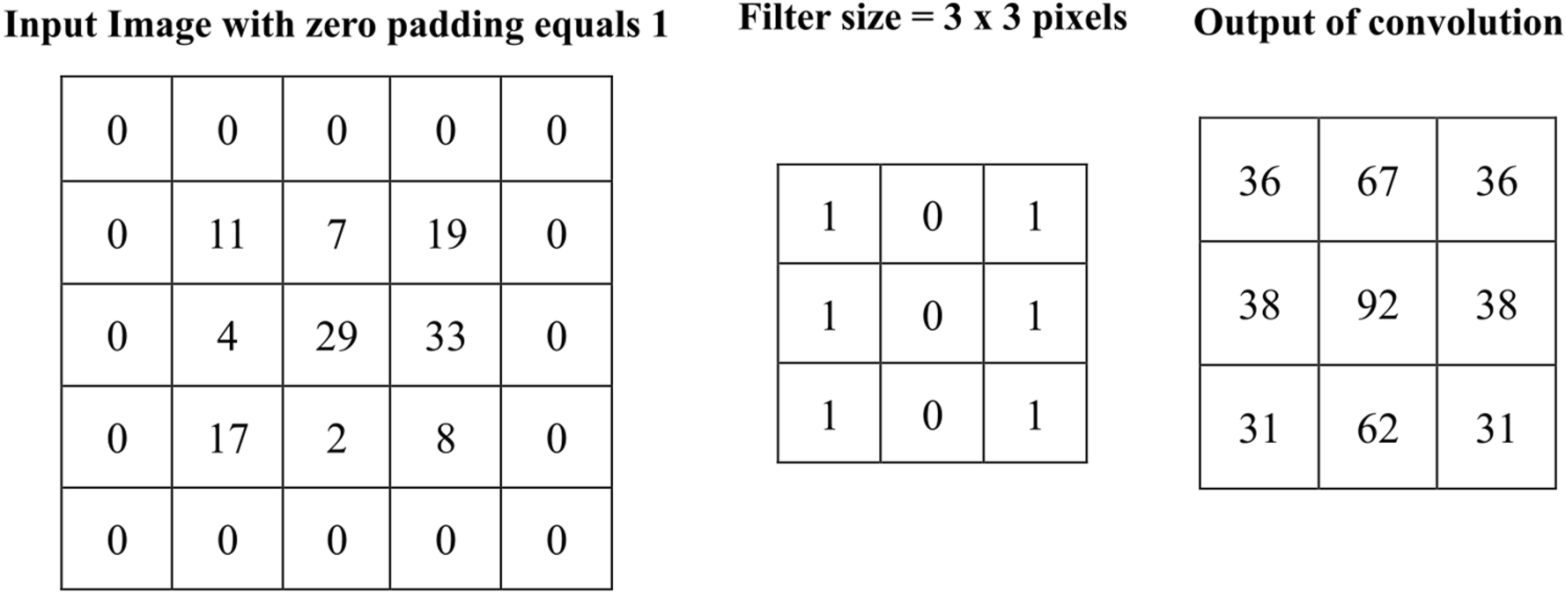
Example of a filter applied to a two-dimensional input image to create a feature map.

In this work, AlexNet CNN model is used to extract DFs from the FCGR images. It is a quick way to use the potential of deep learning without investing time and effort in training the full AlexNet CNN model, which only requires a single pass through the training images^44,45^. AlexNet is a pre-trained CNN model developed by Krizhevsky et al.^46^. It has been trained on over a million images from the ImageNet dataset to categorize them into 1000 classes. Table 3 presents the complete structure of the AlexNet model. The following explains how the AlexNet model extracts DFs and performs the classification process. The AlexNet model consists of 25 deep layers, as shown in Table 3. The convolutional layers compute the product between the input image (227 × 227 × 3) and the K convolution filters of size (N x N x M). These filters move over the image with horizontal and vertical steps known as a stride, as shown in Fig. 4. The filters serve as feature identifiers, with the early layer filters detecting low-level features and the advanced layer filters detecting complex features^46–49^. Figure 5 shows the result of applying a filter of size (3 × 3 pixels) over an input image of size (3 × 3 pixels) with a stride of size (1 × 1 pixel).

**Table 3:** Pre-trained AlexNet model architecture.

Layer name	Input size	Number of kernels	Size of kernels	Stride	Padding	Output size
‘data’	227 × 227 × 3	–	–	–	–	227 × 227 × 3
‘conv1’	227 × 227 × 3	96	11 × 11 × 3	4	0	55 × 55 × 96
‘relu’	55 × 55 × 96	–	–	–	–	55 × 55 × 96
‘norm1’	55 × 55 × 96	–	–	–	–	55 × 55 × 96
‘pool1’	55 × 55 × 96	–	3 × 3	2	0	27 × 27 × 96
‘conv2’	27 × 27 × 96	2 × 128	5 × 5 × 48	1	2	27 × 27 × 256
‘relu2’	27 × 27 × 256	–	–	–	–	27 × 27 × 256
‘norm2’	27 × 27 × 256	–	–	–	–	27 × 27 × 256
‘pool2’	27 × 27 × 256	–	3 × 3	2	0	13 × 13 × 256
‘conv3’	13 × 13 × 256	384	3 × 3 × 256	1	1	13 × 13 × 384
‘relu3’	13 × 13 × 384	–	–	–	–	13 × 13 × 384
‘conv4’	13 × 13 × 384	2 × 192	3 × 3 × 192	1	1	13 × 13 × 384
‘relu4’	13 × 13 × 384	–	–	–	–	13 × 13 × 384
‘conv5’	13 × 13 × 384	2 × 128	5 × 5 × 48	1	1	13 × 13 × 256
‘relu5’	13 × 13 × 256	–	–	–	–	13 × 13 × 256
‘pool5’	13 × 13 × 256	–	3 × 3	2	0	6 × 6 × 256
‘fc6’	6 × 6 × 256	–	–	–	–	1 × 1 × 4096
‘relu6’	1 × 1 × 4096	–	–	–	–	1 × 1 × 4096
‘drop6’	1 × 1 × 4096	–	–	–	–	1 × 1 × 4096
‘fc7’	1 × 1 × 4096	–	–	–	–	1 × 1 × 4096
‘relu7’	1 × 1 × 4096	–	–	–	–	1 × 1 × 4096
‘drop7’	1 × 1 × 4096	–	–	–	–	1 × 1 × 4096
‘fc8’	1 × 1 × 4096	–	–	–	–	1 × 1 × 1000
‘prob’	1 × 1 × 1000	–	–	–	–	1 × 1 × 1000
‘output’	1 × 1 × 1000	–	–	–	–	–

**Figure 6:**
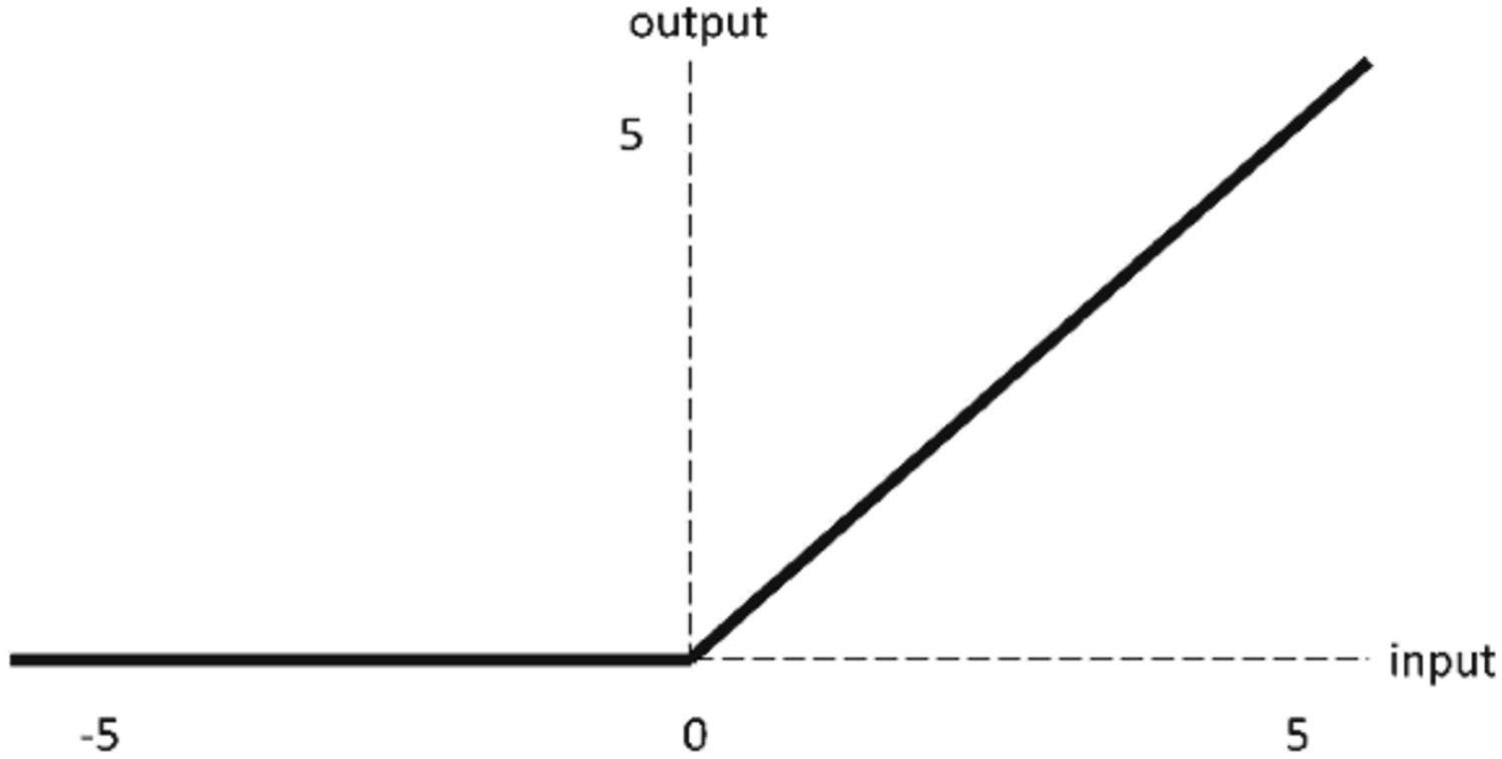
The ReLU activation function.

**Figure 7:**
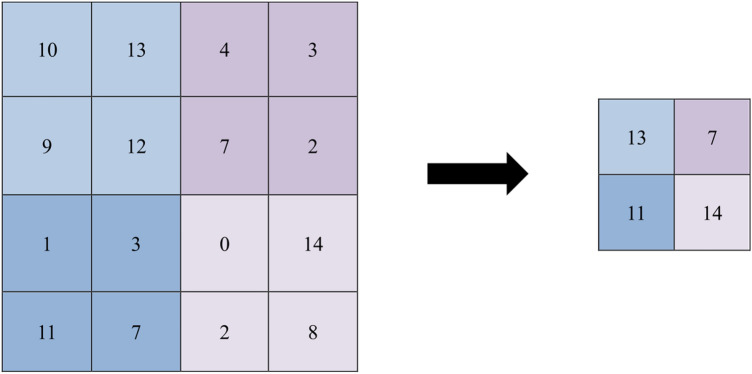
The output of max pooling for an image of size 4 × 4 pixels, a window of size 2 × 2 pixels, and a stride of 2 × 2 pixels.

ReLU layers follow every convolutional and fully-connected layer to reduce the training time. The ReLU activation function of the AlexNet model works in such a way that; when the input is positive, the output equals the input. Otherwise, the output is 0, as shown in Fig. 6. Cross-channel normalization layers follow the first and second ReLU layers, which use local responses in different channels to normalize the input layer by scaling and adjusting the related activations. The normalization can be used in backpropagation and network training acceleration. The AlexNet model uses five channels per element. Maximum pooling layers follow the cross-channel normalization layers and the fifth convolutional layer. The maximum pooling layer divides the entire image into small rectangles, moves these rectangles over the image with a predetermined step, and takes only the maximum value of the elements, as shown in Fig. 7. Consequently, it is used for downsampling to achieve spatial invariance and reduce the number of parameters and computations in the model^46–49^. The pooling layer of the AlexNet has a window size of 3 × 3 pixels with a stride of 2 × 2 pixels.

A dropout layer follows the first and second fully-connected layers to reduce overfitting, where some activations are dropped out randomly, which significantly helps in reducing the training time. The output of the last fully-connected layer is 1000 classes. A SoftMax layer follows the last fully-connected layer to squash all the predicted classes between 0 and 1, such that the total sum of these values equals 1. Finally, the classification output layer of the AlexNet model uses the cross-entropy loss as a cost function to estimate the classification loss and assign a predicted label to each input image^46–49^. The classification loss is estimated using the following equation^49^:

6 $$H \left(p,q\right)=-\sum_{x \in c}{\varvec{p}}\left({\varvec{x}}\right)\times \mathrm{log}{\varvec{q}}\left({\varvec{x}}\right)$$

where ***p*(*x*)** is the target label vector, c is the number of classes, and ***q*(*x*)** is the predicted vector of the SoftMax layer.

#### Stochastic gradient descent optimization algorithm

The cost function evaluates the model's performance concerning predicted and actual outputs. Deep learning optimization algorithms seek to minimize the cost function of the model, thus allowing for better prediction. As a result, selecting the appropriate optimization algorithm and comprehending the function of its parameters allows for targeted fine-tuning of the hyperparameters to produce an effective predictive model. The gradient descent (GD) algorithm minimizes the model's error (cost function) by modifying its parameters until reaching the minimum error value (best model). The model's parameters represent the weights of the model. The size of the steps taken by the GD algorithm to reach the global minimum is determined by the learning rate parameter. There are three types of GD: batch, mini-batch, and stochastic. They vary in the number of training examples used to calculate the model's error and update its parameters (weights)^50^.

The Stochastic GD (SGD) optimization algorithm computes the gradient of the error (cost function) with respect to the model’s parameters and then updates the model’s parameters for each training sample ${x}^{\left(i\right)}$ and label ${y}^{\left(i\right)}$ in the training dataset using Eq. (7). In other words, it uses only one example to take a single step. The SGD algorithm minimizes the cost function of the AlexNet model by updating the model parameters to reach the global minimum (best model) by taking small steps to the opposite gradient direction of the cost function with respect to the model parameters. The AlexNet model uses the SGD algorithm to find the optimum parameters (weights) that correspond to the best fit between predicted and actual outputs^50^.

7 $$\theta =\theta -{\eta \nabla }_{\theta } J\left(\theta ;{x}^{\left(i\right)};{y}^{\left(i\right)}\right),$$

where $\theta$ represents the weight of the model, $\upeta$ is the learning rate, and ${\nabla }_{\theta }$ is the gradient of the cost function $J\left( {\theta ;x^{\left( i \right)} ;y^{\left( i \right)} } \right)$ with respect to the model’s parameters.

Recall that the AlexNet model requires the input image to be in the dimension of 227 × 227 × 3. Accordingly, the FCGR images were resized from 256 × 256 pixels to 227 × 227 pixels. RGB images have three color channels: red, green, and blue, through grayscale images have only one channel. Therefore, the single channel of the FCGR images was replicated to create the input structure (RGB) required by the AlexNet model. The DFs were extracted using the last convolution layer, the conv5 layer, and the second fully-connected layer, the fc7 layer. The conv5 layer resulted in low-level features (256), while the fc7 layer resulted in high-level features (4096). These vectors were fed to KNN and DT classifiers to detect COVID-19, among other HCoV diseases. The AlexNet model was chosen because it requires a short time to extract DFs from its layers. In addition, it results in high performance with medical images for detecting different diseases. In this work, it entailed just 284.34 and 302.55 s to extract DFs from the conv5 and fc7 layers, respectively.

### Feature selection

Feature selection is a crucial stage in machine learning, which extracts the best attributes needed for the classification process. Feature selection approaches have many advantages; they reduce the system training time, improve accuracy, and eliminate overfitting problems^51^. This study used the ReliefF and LASSO algorithms to select the significant features from the DFs of the conv5 and fc7 layers to detect COVID-19, among other HCoV diseases, with high accuracy.

#### ReliefF algorithm

The Relief algorithm is a supervised feature selection approach developed by Kira and Rendell^52^. It depends on the filter-method approach to rank the features to select the most important ones. The basic principle of the original Relief algorithm is to estimate the weight (*w*) of each feature (*f*) in the feature vectors (conv5 and fc7). The essential features have high weights, whereas the redundant ones have small weights. These weights, which range from 1 (best) to − 1 (worst), can be ranked to select the top-scoring features used in the classification tasks^52–55^. The Relief algorithm works as follows. It selects a random observation (*R*) and searches for its two nearest neighbors (*k* = 2): one in the same class, called the nearest hit (*H*), and the other in the opposite class, called the nearest miss (*M*). Then, it modifies the weight of each feature ***w[f]*** based on the feature values of the *R*, *M*, and *H* observations. If the difference between the *R* and *H* for feature (*f*) is high (significant difference), the feature cannot be used to distinguish between the different classes, and its weight ***w[f]*** is reduced. However, if there is a significant difference between the *R* and *M* for feature (*f*), the feature may be used to distinguish between the different classes, and its weight ***w[f]*** is increased. This process will be continued for n times, a user-selected parameter. The pseudo-code of the Relief algorithm is represented as follows:
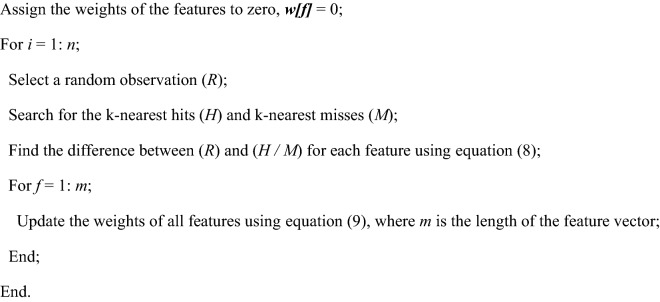


The ReliefF algorithm is an extended version of the Relief algorithm. It searches for the *k*-nearest hits and misses instead of the two nearest neighbors (hit and miss). The selected value of *k* in this study was 10^55^. Initially, the weight of each feature *(f)* in the vector was set to zero (***w[f]*** = 0). Then, an *R* was drawn from the HCoV dataset, and the *k*-nearest hits (*H*_*J*_) and misses (*M*_*J*_) were found using the Manhattan distance. The difference in the value of each feature *(f)* was calculated between *S1* and *S2* using Eq. (8), where *S1* represents an *R*, and *S2* represents the nearest hit (*H*_J_) or miss (*M*_J_). Finally, the weight of each feature was updated using Eq. (9) based on the values obtained from Eq. (8). This process is repeated *n* times, where *n* is the number of training observations in the HCoV dataset^53,54^.

8 $$diff \left( {f, S1, S2} \right) = \frac{{\left| {value\left( {f, S1} \right) - value\left( {f, S2} \right)} \right|}}{max\left( f \right) - min\left( f \right)},$$

where *diff* represents the difference value, *f* represents a feature, *S1* represents a random observation, *S2* represents the nearest hit (*H*_*J*_) or miss (*M*_*J*_), and *max(f)*_*J*_ and *min(f)* represent the maximum and minimum values of each feature.

9 $${\varvec{w}}\left[ {\varvec{f}} \right] : = {\varvec{w}}\left[ {\varvec{f}} \right] - \mathop \sum \limits_{J = 1}^{k} \frac{{diff \left( {f,R,H_{J} } \right)}}{n \times k} + \mathop \sum \limits_{J = 1}^{k} \frac{{diff \left( {f,R,M_{J} } \right)}}{n \times k},$$

where ${\varvec{w}}\left[{\varvec{f}}\right]$ represents the weights of the feature vector**,** *n* is the number of training observations, ${H}_{J}$ and ${M}_{J}$ represent the Jth observations from the same and opposite classes concerning the random observation (*R*), respectively, and *k* is the number of the nearest hits and misses.

The weights of the features were ranked from the best to the worst value to select the most significant features. These features were selected with a start of 10 and a step of 10 until the last element of the conv5 (256) and fc7 (4096) feature vectors. The selected features were fed to the KNN and DT classifiers to evaluate their efficiency in detecting COVID-19, among other HCoV diseases.

#### Least absolute shrinkage and selection operator algorithm

The fundamental objective of linear regression is to find an equation that can predict the dependent variable ($p$) given the independent variables (*f*). The dependent variable is called the response or predicted variable (label). Independent variables are called predictor or explanatory variables (input features). This equation is given as follows:

10 $${\varvec{p}}_{{\varvec{i}}} = { }\upbeta_{0} + \upbeta_{1} f_{i1} + \upbeta_{2} f_{i2} + \cdots + \upbeta_{m} f_{im} = \upbeta_{0} + \mathop \sum \limits_{j = 1}^{m} {\varvec{\beta}}_{{\mathbf{j}}} {\varvec{f}}_{{\user2{ij }}} ,$$

where ${{\varvec{p}}}_{{\varvec{i}}}$ is the label at observation *i*, ${\upbeta }_{0}$ is the intercept of *p*, ${{\varvec{f}}}_{{\varvec{i}}{\varvec{j}}}$ is the jth feature at observation *i*, ${\upbeta }_{\mathrm{j}}$ is the coefficient of the jth feature, and *m* is the number of features.

The linear regression selects coefficients (β) for each independent variable (*f*) that minimizes the cost function given by Eq. (11), which is the mean squared error (MSE) between the actual and predicted outputs.

11 $${\text{MSE}}\left( {\upbeta } \right) = \mathop \sum \limits_{i = 1}^{n} \left( {{\varvec{a}}_{{\varvec{i}}} - {\varvec{p}}_{{\varvec{i}}} } \right)^{2} ,$$

where *n* is the number of training observations, ${{\varvec{a}}}_{{\varvec{i}}}$ and ${{\varvec{p}}}_{{\varvec{i}}}$ are the actual and predicted outputs at observation i.

The linear regression does not remove any features from the subset. It gives weight to each feature. However, the LASSO regression proposed by Robert Tibshirani^56^ removes the less significant features from the subset. The LASSO modifies the cost function of the linear regression by adding a regularization parameter ($\uplambda )$ that penalizes the absolute sum of all coefficients to minimize the MSE, which is given by the following equation^56,57^:

12 $${\text{MSE}}\left( {\upbeta } \right) = \mathop \sum \limits_{i = 1}^{n} \left( {{\varvec{a}}_{{\varvec{i}}} - {\varvec{p}}_{{\varvec{i}}} } \right)^{2} + {\uplambda }\mathop \sum \limits_{j = 1}^{m} \left| {{{\varvec{\upbeta}}}_{{\mathbf{j}}} } \right|,$$

where λ is a nonnegative regularization parameter, and ${{\varvec{\upbeta}}}_{\mathbf{j}}$ is a coefficient vector of length *m*.

As $\uplambda$ increases, the regularization strength increases; therefore, the absolute values of weights would need to decrease (shrink) to keep the value of the cost function minimized. Therefore, the LASSO results in the less significant weights becoming zero, and their features are removed from the subset. As a result, LASSO regression has the significant advantage of performing an automatic feature selection. The LASSO heavily relies on the lambda (λ), which is the controlling factor in shrinkage. All features are considered when λ equals zero. As λ increases, the number of selected features decreases; whereas λ approaches infinity, all features are removed from the subset. Suppose there is a high correlation between a group of features. In that case, their presence will raise the cost function value, so LASSO selects one from them and shrinks the coefficient of the others to zero to select the best feature subset^56–59^.

The LASSO algorithm was implemented with different λ values with indices ranging from the 1st to the 100th order using the tenfold cross-validation strategy to detect the best value of λ that minimizes the cost function and results in the most significant features among the DFs of the conv5 (256) and fc7 (4096) layers. These features were fed to the KNN and DT classifiers to evaluate their efficiency in detecting COVID-19, among other HCoV diseases.

### Classification process

**Figure 8:**
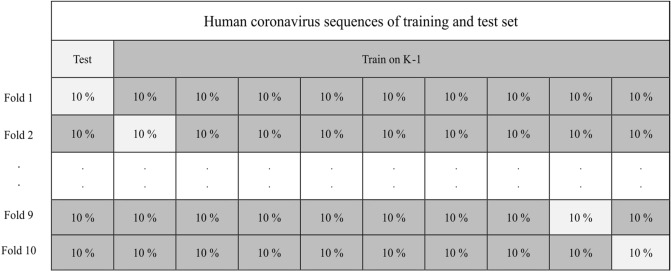
Tenfold cross-validation technique.

The most significant features obtained using the ReliefF and LASSO algorithms were employed to train and test the KNN and DT classifiers to detect COVID-19, among other HCoV diseases. Several *k *values (1, 3, 5, 7, and 9) were used to implement the KNN. The performance of the hybrid approach slightly decreased for *k* above 3. As a result, the *k* value was set to 3. A tenfold cross-validation strategy was used to evaluate the performance of the hybrid deep learning approach, such that all HCoV sequences were used for training and testing, as shown in Fig. 8. The average of 20 runs was used to report the results of the study. Each classifier was trained and tested using the different selected features obtained using the ReliefF and LASSO algorithms to detect the optimum approach that provides the best performance with a minimum number of selected features.

### System evaluation

Some effective parameters were used to evaluate the performance of the proposed hybrid deep learning approach. These parameters are given by the following equations^60^:

13 $${\text{Accuracy}} = \frac{Tp + Tn}{{Tp + Fp + Tn + Fn}} \times 100\% ,$$

14 $${\text{Precision}} = \frac{Tp}{{Fp + Tp}} \times 100\% ,$$

15 $${\text{Specificity}} = \frac{Tn}{{Tn + Fp}} \times 100\% ,$$

16 $${\text{Sensitivity}} = \frac{Tp}{{Tp + Fn}} \times 100\% ,$$

17 $${\text{F}}_{1} {\text{-score}} = \frac{2Tp}{{2Tp + Fp + Fn}},$$

18 $${\text{Matthew's correlation coefficient}}\;\left( {{\text{MCC}}} \right) = \frac{{\left( {Tp \times Tn} \right) - \left( {Fn \times Fp} \right)}}{{\sqrt {\left( {Tp + Fp} \right)\left( {Fp + Tn} \right)\left( {Tn + Fn} \right)\left( {Fn + Tp} \right)} }},$$

where *Fn* and *Tn* denote false-negative and true-negative values, and *Fp* and *Tp* denote the false-positive and true-positive values, respectively.

### Hyperparameter tuning

The hyperparameters of the proposed technique are represented in the feature selection algorithms (ReliefF and LASSO) and the machine learning classifiers (KNN and DT). Since the pre-trained AlexNet model is employed as a feature extractor, it is implemented using its pre-trained weights to extract DFs from the conv5 and fc7 layers. For the ReliefF algorithm, the number of the k-nearest hits and misses was 10. The procedure of the ReliefF algorithm was repeated by the number of training samples (90% of the HCoV dataset). After applying the ReliefF algorithm, the features were ranked from the highest to the lowest based on their weights. Then, the best features were selected with a start of 10 and a step of 10 until the last element of the feature vectors; 256 for the conv5 layer and 4096 for the fc7 layer. The number of selected features that resulted in the best performance was 200 and 550 for the conv5 (256) and fc7 (4096) layers. For the LASSO algorithm, the regularization parameter ($\uplambda )$ was applied with values ranging from the 1st to the 100th order to find the best order that minimizes the cost function (MSE). The best order for the DFs of the conv5 layer was the fifth order ($\uplambda$ = 3.71 × 10^–5^), while for the DFs of the fc7 layer was the twelfth order ($\uplambda \hspace{0.17em}$= 8.51 × 10^–5^).

The neighborhood size (k) of the KNN classifier was selected with values from 1 to 10 with a step of 2 (binary classification). It was observed that the performance slightly decreased with increasing the value of *k* above 3. Therefore, the *k* value was set to 3. In the prediction process of the KNN classifier, the class to which a new observation data belongs is determined by calculating the shortest euclidean distance metric between the observation sample and its k-nearest neighbor samples because it outperformed the other distance metrics. For the DT classifier, the values of the maximum splits, minimum leaf size, and maximum leaf size were 100, 1, and 10, respectively. Table 4 summarizes the best values of the hyperparameters of the proposed hybrid deep learning approach.

**Table 4:** The hyperparameters of the proposed technique.

Hyperparameter	Best value
ReliefF algorithm	
K-nearest hits and misses	10
N	6635
Number of selected features (conv5)	200
Number of selected features (fc7)	550
LASSO algorithm	
$\uplambda$(conv5 features)	3.71 × 10^–5^
$\uplambda$(fc7 features)	8.51 × 10^–5^
KNN classifier	
Number of neighbors	3
Distance metric	Euclidean distance
DT classifier	
Maximum splits	100
Minimum leaf size	1
Maximum leaf size	10

## Results and discussion

### Proposed approach results

The proposed approach was implemented using MATLAB-R2020a software installed on a laptop with a 2.5 GHz Intel Core i5 CPU and 16 GB RAM. Table 5 shows the time required to extract DFs from the conv5 and fc7 layers of the AlexNet CNN model. The low-level features extracted from the conv5 layer require a slightly shorter time than the high-level features extracted from the fc7 layer. Generally, the AlexNet took a short time, less than 303 s, to extract DFs from its last layer. Consequently, the potential of deep learning was used to quickly extract DFs to rapidly detect COVID-19, among other HCoV diseases, with high acceptable accuracy.

**Table 5:** Time required for feature extraction from the conv5 and fc7 layers.

Layer name	Time of feature extraction (s)
conv5 (256 features)	284.39
fc7 (4096 features)	302.56

**Figure 9:**
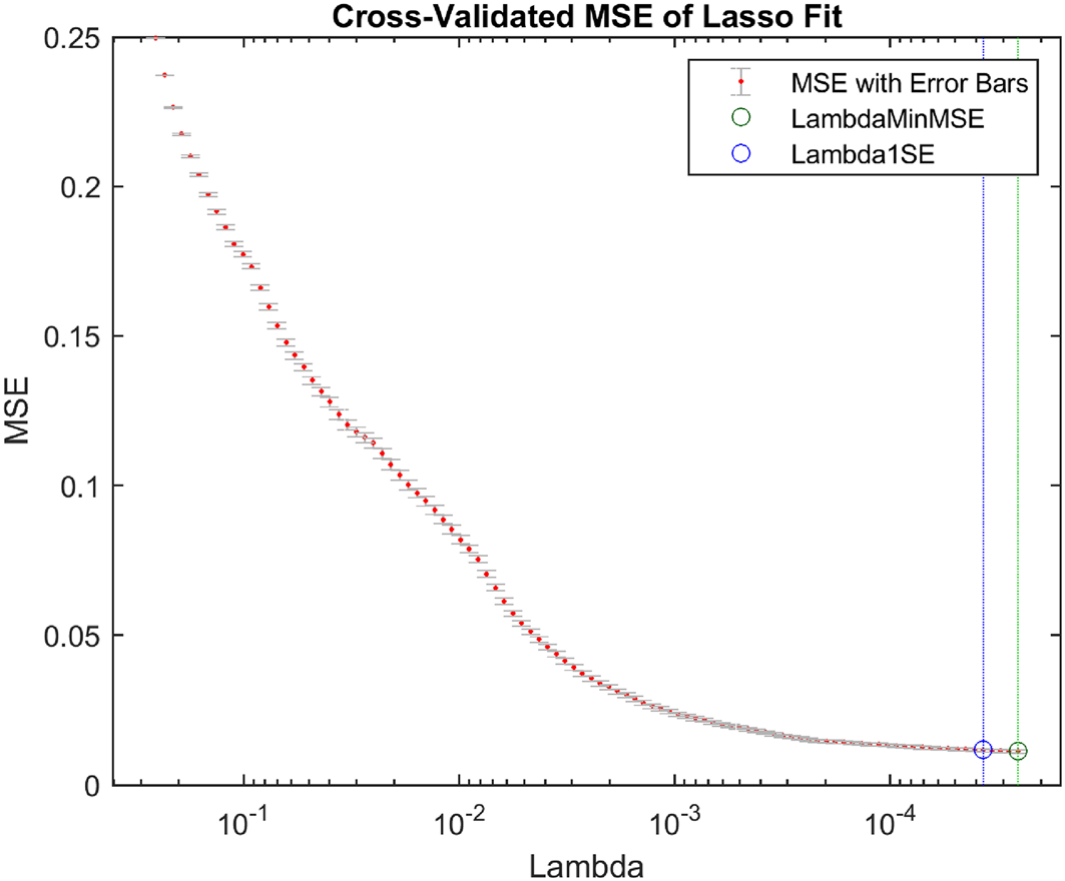
The LASSO fit using the DFs of the conv5 layer.

**Figure 10:**
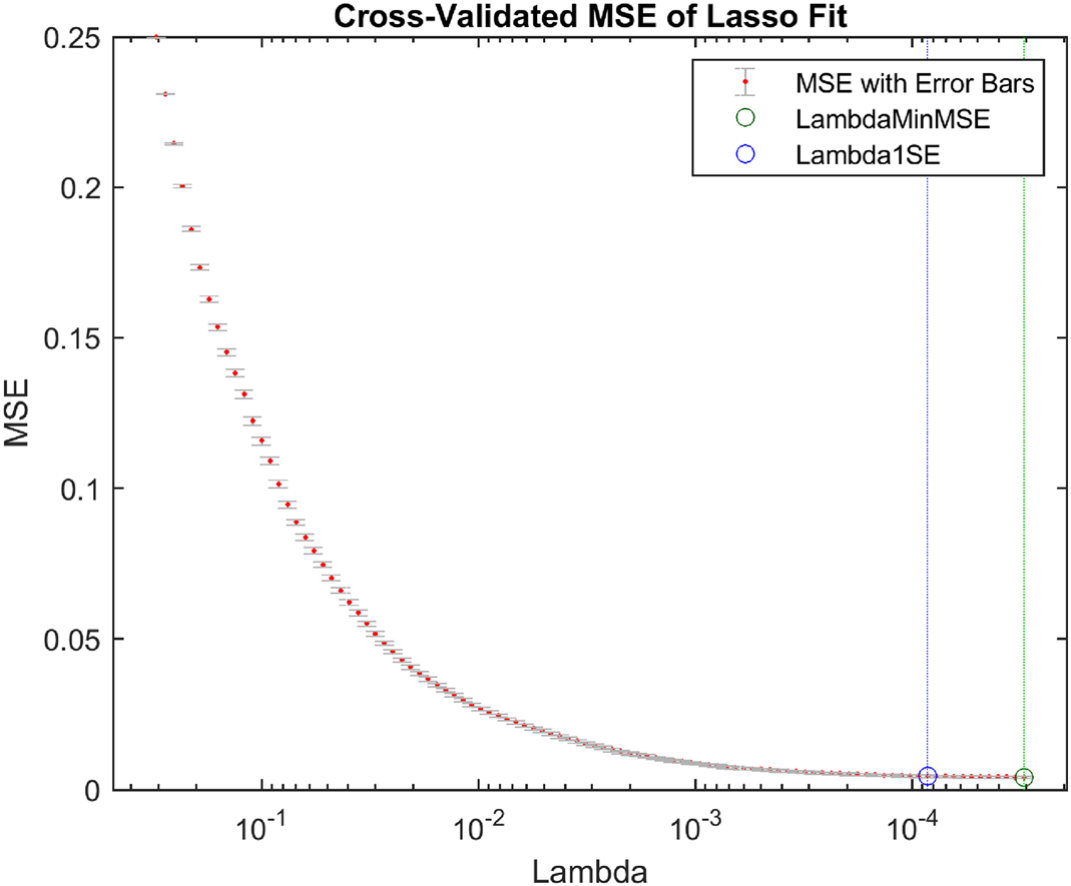
The LASSO fit using the DFs of the fc7 layer.

Table 6 shows the performance of the KNN and DT classifiers using the DFs directly without feature selection. The research findings showed that the DFs of the fc7 layer resulted in slightly higher performance than that of the conv5 layer. The KNN accuracy was 99.59% with the fc7 layer compared with 99.53% with the conv5 layer. The DT accuracy was 98.36% with the fc7 layer compared with 98.07% with the conv5 layer. The KNN–fc7 approach provided the best performance, with 99.59% accuracy with an execution time of 2960.9 s to train and test the system. Figures 9 and 10 show the cross-validated MSE of the LASSO fit using the DFs of the conv5 and fc7 layers, respectively. From Figs. 9 and 10, the DFs of the fc7 layer resulted in an MSE less than that of the conv5 layer. The MSE of the fc7 layer was 0.00453 compared to 0.0117 for the conv5 layer. The lambdas that provided the minimum MSE for the DFs of the conv5 and fc7 layers were 3.71 × 10^–5^ and 8.51 × 10^–5^, respectively. The number of significant features obtained using these lambdas was 112 and 437 for the conv5 and fc7 layers, respectively.

**Table 6:** Performance of the hybrid approach using the deep features without feature selection.

Approach	Accuracy (%)	Precision (%)	Sensitivity (%)	Specificity (%)	F_1_-score	MCC	No. of features	Classification time (s)
conv5–KNN	99.53	99.62	99.44	99.63	0.995	0.991	256	190.97
conv5–DT	98.07	98.50	97.65	98.49	0.981	0.961	256	161.54
fc7–KNN	99.59	99.69	99.51	99.69	0.996	0.992	4096	2960.9
fc7–DT	98.36	98.41	98.35	98.39	0.983	0.967	4096	4083.5

As shown in Tables 7 and 8, the ReliefF and LASSO algorithms improved the performance of COVID-19 detection with a few selected features instead of using the total DFs in the classification process. The ReliefF–KNN approach increased the COVID-19 detection accuracy for conv5 and fc7 layers from 99.53% and 99.59% to 99.55% and 99.67%, using only 200 and 550 DFs instead of 256 and 4096 DFs, respectively. However, the LASSO–KNN approach resulted in higher performance parameters than the ReleifF–KNN approach, with only 112 and 437 DFs. A similar finding was obtained with the DT classifier, with better evaluation parameters’ values for LASSO than for ReleifF. Therefore, the LASSO algorithm outperformed the ReliefF algorithm regarding the system performance and the number of selected features used in the classification process. Besides these advantages, the LASSO algorithm took a little time to select the most significant features (fc7 layer) compared with the ReliefF algorithm, as shown in Table 9. The results shown in Table 9 were obtained by running the ReleifF and LASSO algorithms 20 times and then reporting the mean values.

**Table 7:** Performance of the hybrid approach using the ReliefF feature selection technique.

Approach	Accuracy (%)	Precision (%)	Sensitivity (%)	Specificity (%)	F_1_-score	MCC	No. of features	Classification time (s)
conv5–KNN	99.55	99.65	99.45	99.65	0.996	0.991	200	174.19
conv5–DT	98.15	98.67	97.63	98.67	0.981	0.963	200	146.30
fc7–KNN	99.67	99.74	99.61	99.74	0.997	0.993	550	406.88
fc7–DT	98.47	98.63	98.33	98.62	0.985	0.969	550	520.04

**Table 8:** Performance of the hybrid approach using the LASSO feature selection technique.

Approach	Accuracy (%)	Precision (%)	Sensitivity (%)	Specificity (%)	F_1_-score	MCC	No. of features	Classification time (s)
conv5–KNN	99.57	99.65	99.47	99.64	0.996	0.991	112	97.25
conv5–DT	98.24	98.75	97.73	98.75	0.982	0.965	112	74.61
fc7–KNN	99.71	99.77	99.62	99.78	0.998	0.995	437	369.04
fc7–DT	98.57	98.59	98.56	98.57	0.986	0.971	437	487.84

**Table 9:** Time of feature selection using the ReliefF and LASSO algorithms.

Layer name	ReliefF (s)	LASSO (s)
conv5 (256 features)	105.09	353.73
fc7 (4096 features)	1529.9	824.36

Table 10 compares the results obtained with and without feature selection algorithms regarding the maximum accuracy, number of selected features, and execution time. The LASSO–KNN approach increased the detection accuracy from 99.59 to 99.71%, using only 437 selected features instead of 4096 features. Also, it reduced the execution time from 3263.16 to 1495.96 s. Generally, the accuracy of the different approaches was above 98%. This demonstrates the efficacy of the AlexNet in extracting DFs from the eighth-order FCGR images and achieving accurate detection of COVID-19, among other HCoV diseases. The KNN classifier resulted in high performance compared with the DT classifier: high values for all evaluation parameters, as shown in Tables 6, 7, and 8. Finally, extracting DFs from the fc7 layer, selecting the significant features with the LASSO algorithm, and executing the classification process using the KNN classifier resulted in the perfect approach that provided high accuracy (99.71%) with a few selected features (437) and a short execution time (1495.96 s). The LASSO–KNN approach detected COVID-19, among other HCoV diseases, with 99.71% accuracy, 99.78% specificity, 99.62% sensitivity, 99.77% precision, 0.995 MCC, and 0.998 F_1_-score.

**Table 10:** Maximum performance of the hybrid approach using the KNN classifier.

Feature type	Maximum accuracy (%)	No. of features	Execution time (s)
Without feature selection	99.59	4096	302.56 + 2960.6 = 3263.16
ReliefF algorithm	99.68	550	302.56 + 1529.9 + 487.84 = 2320.03
LASSO algorithm	99.71	437	302.56 + 824.36 + 369.04 = 1495.96

#### Comparison with the earlier COVID-19 studies

In this section, the proposed system is compared with the earlier studies that used genome sequences to detect COVID-19^26–35,38,39^. The results of all reviewed studies are summarized in Table 11. Arslan et al.^26^ and Arslan^27^ presented systems for detecting COVID-19 by extracting standard features from whole genome sequences. Their system achieved remarkable accuracy (above 98.4%) but had certain limitations. Arslan et al.^27^ used a dataset that eliminated the SARS-CoV-1 sequences, which have high similarity (79%) to the COVID-19 sequences^8^. Lopez-Rincon et al.^28^ presented a deep-learning approach to detect COVID-19 using whole genome sequences. The system achieved high accuracy (98.73%), but their dataset was imbalanced with just 66 COVID-19 sequences compared with 478 other sequences. As a result, there was relatively little information about the COVID-19 class. Thus, their system could not distinguish rare COVID-19 sequences from the majority.

**Table 11:** Results of the earlier COVID-19 studies.

Study	Best technique	Dataset	Maximum accuracy (%)
^26^	CpG features KNN classifier	COVID-19: 1000 HCoV-HKU1: 18 HCoV-NL63: 61 MERS-CoV: 258 βCoV: 140 HCoV-229E: 27	98.4
^27^	CpG and similarity features KNN classifier	COVID-19: 1000 HCoV-HKU1: 27 HCoV-NL63: 64 MERS-CoV: 339 HCoV-OC43: 145 SARS-CoV-1: 12 HCoV-229E: 28	99.8
^28^	Convolution neural network model	COVID-19: 66 Others: 487	98.73
^29^	Recurrent neural network	COVID-19: 680 Influenza: 8576	99.51
^30^	Chaos features SVM classifier	COVID-19: 4498 SARS-COV-1:101	99
^31^	Deep features MLP classifier	COVID-19:171 Influenza: 347,162	98
^32^	Standard features extracted from genomic signals KNN classifier	SARS-CoV-1: 76 MERS-CoV: 76 COVID-19: 76	100
^33^	DFT features extracted from genomic signals Linear discriminant analysis classifier	COVID-19: 29 αCoV: 20 βCoV: 20 δCoV: 20	100
^34^	Linear predictive coding model SVM classifier	COVID-19:47,200 Influenza: 59,800	99.4
^35^	Standard features extracted from genomic signals Random forest classifier	COVID-19: 615 Coronavirus: 967	97.4
^38^	Standard features extracted from single nucleotide gray-level representation images KNN classifier	SARS-CoV-1: 57 MERS-CoV: 258 COVID-19: 300	100
^39^	Standard features extracted from fourth-order FCGR images KNN classifier	COVID-19: 3700 Coronavirus: 3663	99.39
Proposed approach	fc7 layer of the AlexNet model Deep features extracted from eight-order FCGR images KNN classifier	COVID-19: 3700 HCoV-HKU1: 412 HCoV-NL63: 637 MERS-CoV: 734 HCoV-OC43: 1351 SARS-CoV-1: 64 HCoV-229E: 465	99.71

*KNN* K-nearest neighbors, *SVM* support vector machine, *MLP* multilayer perceptron classifier, *DFT* discrete Fourier transform, *FCGR* frequency chaos game representation.

Saha et al.^29^ and Harikrishnan et al.^30^ presented binary systems that achieved high accuracy. However, both systems suffer from an imbalance problem in their dataset, especially the dataset of Harikrishnan^30^, which included 4498 COVID-19 sequences and 101 SARS-CoV-1 sequences. Gomez et al.^31^ identified COVID-19 among 23 virus families. The main limitation of their system was the small number of COVID-19 cases (171). In addition, their system excluded the SARS-CoV-1 and MERS-CoV sequences, which have a genetic similarity of about 79% and 50% with COVID-19 sequences^8^.

Naeem et al.^32^ proposed a GSP approach to classify COVID-19, SARS, and MERS diseases. Although their system achieved 100% performance, it had many limitations. The dataset size was small, including only 76 sequences for each disease. In addition, they evaluated the system using the train and test split approach, which only provides accurate results with a large dataset. Finally, the dataset included whole genome sequences of only three types of HCoV diseases, limiting their system in analyzing the other types of HCoV diseases. Randhawa et al.^33^ presented an approach to classify COVID-19 under three genera: αCoV, βCoV, and δCoV. Their system resulted in 100% accuracy. The limited number (20 for each type) of genome sequences used in their dataset is the main limitation of their approach. Therefore, the approach accuracy may decrease when the number of genome sequences increases. Furthermore, their dataset includes δCoVs that mostly infect bird species instead of humans. Additionally, they performed classification at the genus level; therefore, their approach may not detect COVID-19 from the other diseases related to the βCoV family, such as MERS and SARS diseases. Therefore, the accuracy of the system may drop if other HCoV sequences that are genetically similar to COVID-19 are added to their dataset.

Khodaei et al.^34^ presented an effective system to classify COVID-19 among influenza cases. The length of the whole genome sequences of COVID-19 and influenza viruses was about 13,000 and 30,000 base pairs. Therefore, detecting COVID-19 from influenza viruses is not a challenging issue. Hammad et al.^38^ presented a GIP system to identify COVID-19, SARS, and MERS diseases. The main limitation of the approach is that the image size created using the SGLR technique depends mainly on the sequence length. Therefore, the approach cannot deal with genome sequences that have differences in length (whole and partial genome sequences). Moreover, the system dataset had the Naeem et al.^32^ limitation of including only three variants of HCoV diseases. Although the previous COVID-19 studies achieved highly significant results, they had limitations related to the analysis of partial genome sequences, where their datasets included only whole genome sequences.

Singh et al.^35^ transformed whole and partial genome sequences of HCoV diseases into genomic signals to detect COVID-19. Their system resulted in an accuracy of 97.5%. Although they used whole and partial genome sequences in their dataset, their study had certain limitations. They used the EIIP technique to convert the whole and partial genome sequences into genomic signals, resulting in genomic signals with different lengths. However, the FCGR technique used in this study resulted in uniform-size images for both whole and partial genome sequences. In addition, their dataset size was small, with 1582 cases compared to 7361 cases in our dataset. In this study, the limitations of the previous COVID-19 studies have been overcome in many ways. First, the dataset utilized in this study contains all variants of HCoV diseases that are genetically similar to COVID-19 with both whole and partial genome sequences, thereby demonstrating the effectiveness and strength of the proposed approach. Second, the FCGR approach was used, transforming the whole and partial genome sequences of HCoV diseases into uniform-size images. Finally, the dataset size was very large, where all available sequences were downloaded and used in this study. In addition, our study's results outperformed the state-of-the-art approaches' results^26–35,38,39^, as revealed in this section.

## Conclusions

This study presents an effective hybrid deep learning approach based on GIP techniques to detect COVID-19, among other HCoV diseases. It achieved high accuracy while avoiding the limitations and drawbacks of earlier COVID-19 detection techniques. Generally, the accuracy of the different algorithms was above 98%. This shows the efficacy of the AlexNet model in extracting DFs from the eighth-order FCGR images and achieving accurate detection of COVID-19, among other HCoV diseases. The experimental results showed that the LASSO and ReliefF feature selection algorithms improved the approach performance with a few selected DFs instead of using the total DFs in the classification process. However, the LASSO algorithm outperformed the ReliefF algorithm regarding the system performance, number of selected features, and execution time.

Moreover, the results demonstrated that the DFs of the fc7 layer resulted in high performance compared with the conv5 layer. As well as, the KNN classifier provided high system performance compared with the DT classifier. The proposed hybrid deep learning approach resulted in the best results of 99.71% accuracy, 99.77% precision, 99.62% sensitivity, and 99.78% specificity. This performance was achieved by extracting DFs from the eighth-order FCGR images using the fc7 layer of the AlexNet model, applying the LASSO feature selection algorithm with λ equal to 8.51 × 10^−5^ to select the most significant features (437), and executing the classification using the KNN classifier with *k* value = 3.

## Supplementary Information

Supplementary Information.

## Data Availability

The genome sequences used in the current study are available in the NCBI repository, https://www.ncbi.nlm.nih.gov/labs/virus, and the accession numbers of these sequences are available as supplementary material.
